# Influence of Teaching Styles on the Learning Academic Confidence of Teachers in Training

**DOI:** 10.3390/jintelligence10030071

**Published:** 2022-09-19

**Authors:** Antonio Granero-Gallegos, Juan Carlos Escaravajal, Ginés David López-García, Raúl Baños

**Affiliations:** 1 Department of Education, University of Almeria, 04120 Almeria, Spain; 2 Health Research Centre, University of Almeria, 04120 Almeria, Spain; 3 Department of Musical, Plastic and Corporal Expression, Faculty of Social and Human Sciences, University of Zaragoza, Campus de Teruel, 44003 Zaragoza, Spain; 4 Faculty of Sport Autonomous, University of Baja California, Tijuana 22390, Mexico

**Keywords:** self-determination, autonomous support, controlling style, higher education

## Abstract

The objective of this research was to analyze the mediation of academic engagement and the satisfaction of basic needs between teaching styles and academic confidence amongst teachers during initial training. The research design was observational, descriptive, cross-sectional, and non-randomized. In total, 920 university students in initial teacher training participated (61.85% women) (*M*_age_ = 24.73; *SD* = 5.34). The scales of interpersonal teaching styles, the satisfaction of basic psychological needs, academic engagement, and academic confidence were used, and a structural equation analysis with latent variables was carried out. The results highlight the important mediating role played by the satisfaction of basic psychological needs between the autonomous support style and the academic confidence of the trainee teachers. This research also highlights the importance, both for teachers and researchers, of using an autonomous support style, as well as the creation of a context for encouraging the satisfaction of basic psychological needs, to promote academic confidence in initial teacher training programs at university.

## 1. Introduction

Adequate teaching preparation and intervention are considered important factors of the educational system (Muñiz-Rodríguez et al. 2016) to face the new challenges and adversities that might emerge in today’s society (Manzano-Sánchez et al. 2021). Accordingly, the teacher training given is especially important for improving the quality of initial teacher training programs (Ping et al. 2018) since it is during this training process that the future teacher can acquire the skills, capabilities, and competencies necessary to address their professional development (Meeus et al. 2018; Muñiz-Rodríguez et al. 2021; Sumantri et al. 2018; Sánchez-Cabrero and Pericacho-Gómez 2022). The demands made during the training process can create negative attitudes, such as a decrease in self-confidence, which might even lead to academic dropout (Demanet and Van Houtte 2019). Conversely, academic confidence is associated with success and an increase in academic performance (Sharma 2016). Furthermore, among the pre-service teachers, it is even associated with the ability to actively involve their future students (Sadler 2013). Therefore, as indicated by the OECD (2014), academic confidence is important in initial teacher training so as to adequately develop the professional skills necessary for future teaching interventions. In addition, this confidence has a significant impact on the students’ desire to learn more in the future (Ireson and Hallam 2009), being closely linked to learning achievement and motivation, both of which are considered factors in self-determination theory (Ryan and Deci 2017). It is also worth noting that this is related to lifelong learning, one of the main objectives of current education, which is considered key to competitiveness and employability at the professional level, including in the teacher training context (OECD 2014). According to the literature, self-confidence is a context-specific variable (Perry 2011) and therefore, authors, such as Eskiler et al. (2012), have shown the need to apply the self-determination theory (SDT; Ryan and Deci 2017) (e.g., taking into account basic psychological needs; BPNs), to determine the self-confidence levels of university students. However, to our knowledge, no recorded studies have examined the effects of the classroom social context generated by the teacher educator, on the academic confidence of the pre-service teachers.

### 1.1. Academic Confidence

Self-confidence is the perception that the individual has of their own skills, aptitudes, and abilities to achieve their goals (see Martins et al. 2018). When a person’s perception of their own abilities is focused on the skills for monitoring and developing classes, and attaining educational goals, we refer to this as academic confidence (Sander and Sanders 2005). In this way, an individual may have great self-confidence on a general level, even if they do not trust their academic abilities. Academic confidence is an important factor of the academic self-concept (ASC) (Granero-Gallegos et al. 2021a; Matovu 2014), as it is one of the important factors in the quality of teaching, since the perceptions that students have about their academic abilities, internalize them and condition certain attitudes (Engels et al. 2004). However, there is abundant scientific literature in the educational context related to student self-confidence (Eskiler et al. 2012; Li et al. 2021; Malandrakis 2018; Martins et al. 2018; Pulford et al. 2018; Sadler 2013, amongst others), with few studies related to academic confidence (Chang and Ye 2022; Granero-Gallegos et al. 2021a). In higher education, research such as that carried out by Malandrakis (2018) has shown that self-confidence is influenced by diverse variables such as prior motivational resources (i.e., basic psychological needs; BPNs) (Eskiler et al. 2012), the level of independence or support received from the teacher (Barlow and McCann 2019) and the cognitive effort for engaging in academic activities (i.e., academic engagement) (Amerstorfer and Freiin von Münster-Kistner 2021; Parker et al. 2017). However, there are few studies that relate academic confidence to variables such as academic engagement (Granero-Gallegos et al. 2021a), and we are not aware of any existing research that has related academic confidence to the interpersonal teaching styles or BPNs. This is evidently a deficit in the scientific literature, since it has recently been demonstrated how important it is for students to develop academic confidence, as this increases their motivation to perform learning tasks and to achieve their academic goals (Chang and Ye 2022; Sander and Sanders 2005) as well as the satisfaction and willingness they feel towards their studies (Bakadorova et al. 2020; Engels et al. 2004). Furthermore, university students with high levels of academic confidence are more predisposed towards the teaching-learning process; they participate more in the learning process itself, are more determined to meet challenges and achieve objectives, and have greater expectations of success, all of which positively affect their academic performance, compared with those with low levels (Akbari and Sahibzada 2020; Pulford et al. 2018). Therefore, to examine the teaching strategies that lead to a greater academic confidence in students, it is important to analyze the mediating roles of prior motivational experiences and academic engagement between the teaching styles and the confidence of pre-service teachers. This is what the present study intends to do. To measure academic confidence, we used the recently validated scale with Spanish university students and it has been shown to be as a valid and reliable scale.

### 1.2. Academic Engagement

Academic engagement has been conceptualized as a positive state of mind characterized by high levels of energy, enthusiasm, and immersion in activities, where time goes by unnoticed (Schaufeli et al. 2006). These authors presented the construct from three main dimensions: (i) vigor (i.e., high energy levels and resilience during study); (ii) dedication (i.e., the full involvement of students in their academic tasks); and (iii) absorption (i.e., high levels of concentration and connection to a task). According to the previous literature, the affective-cognitive involvement of teachers during their initial training process has adaptive consequences on academic results (Kahu 2013), which may increase the perceived self-assessments of their ability to overcome educational demands (i.e., academic confidence) (Amerstorfer and Freiin von Münster-Kistner 2021; Parker et al. 2017). Likewise, studies such as those by DeVito (2016) or Adeshola and Agoyi (2022) have shown that engagement is influenced by various affective, contextual, and motivational factors that are important during the teacher training process (Buzzai et al. 2021; Chan et al. 2021). In adolescent students, the positive influence of the satisfaction of basic psychological needs (SBPNs) on academic engagement has been proven (Buzzai et al. 2021), as well as the positive effect of autonomous support on engagement through the SBPNs (Jang et al. 2016). Therefore, in the university context (i.e., pre-service teachers), analyzing the mediating role of engagement between these prior variables and academic confidence, may be of interest. We choose to select the scale by Schaufeli et al. (2002) to measure academic engagement because it is a contrasted instrument, and the authors offer items for student in Spanish.

### 1.3. Satisfaction of Basic Psychological Needs

BPNs are important variables in explaining the relationship between teaching styles, engagement, and academic confidence. They are also part of the self-determination theory (SDT; Ryan et al. 2021), one of the psychological theories that explains motivational resources in the domain of any human activity. This theory postulates that motivational resources are supported by the SBPNs experiences, understanding them to be essential and universal nutrients for optimal development, growth, and well-being (Vansteenkiste et al. 2020). The literature explains the existence of four basic psychological needs (BPNs): autonomy, relatedness, competence, and novelty (González-Cutre et al. 2020; Ryan and Deci 2017). Autonomy refers to the volition and the decisive capacity that the individual has over their own actions. Competence refers to the desire to feel effective and capable in the activities that are carried out. Relatedness manifests itself as feeling accepted by and connected to others. Novelty is conceptualized as the need to experience something that has not been experienced before. Likewise, the SDT postulates that the SBPNs stimulates the individual’s cognitive, affective, and behavioral consequences in an adaptive way. In the non-university educational field, some studies have shown the importance of the mediating role of the SBPNs between teaching intervention and variables such as engagement (Buzzai et al. 2021; Gao et al. 2021) and self-confidence (Eskiler et al. 2012), evidencing a positive influence. In addition, with regards to university students, some research has shown that the SBPNs are positively influenced by a democratic teaching style (Burgueño et al. 2021) or autonomous support (Reeve et al. 2018), while the influence on the SBPNs of an autocratic style is negative (Burgueño et al. 2021). To the best of our knowledge, we have not found studies that analyze the relationship between the SBPNs and academic confidence, and few studies have related the SBPNs with the ASC (remembering that academic confidence is an important factor in the ASC). In this line, the importance of the SBPNs in primary school students has been demonstrated since, when teachers design classes around the concept of the SBPNs, the ASC increases (Gilbert et al. 2022). Furthermore, when primary students see their BPNs satisfied by their parents, it also improves their ASC (Lu et al. 2017), as measured by the ASC in both studies in a unidimensional way. Despite the findings of previous research conducted on primary school students, to date, no studies have been found that examine this relationship in a teacher education context. Therefore, as Sadler (2013) states, it is important to develop academic confidence in pre-service teachers, as they will be able to pass on these skills to their future students. Consequently, it is interesting to analyze the mediating role of the SBPNs between the interpersonal teaching style and confidence in the pre-service teacher. We used the SBPNs scale of León et al. (2011) with the novelty scale by González-Cutre et al. (2020); the items of the novelty scale were integrated into SBPNs scale as suggested by the authors themselves.

### 1.4. Interpersonal Teaching Style

The research developed within the SDT framework considers that the teaching interaction styles can transform the students’ behavior pattern, thus modifying its adaptive consequences (Ryan and Deci 2017). The style is the interpersonal and behavioral tone that teachers use to involve their students in learning activities (Reeve et al. 2019; Reeve et al. 2018). The SDT proposes the existence of at least two types of teaching styles in teacher and student interactions, namely the autonomous support style and the controlling style (Ryan et al. 2021). The autonomous support style refers to those teaching strategies that identify the students’ interests, justify task explanations, and provide opportunities to choose from (Reeve et al. 2022). In contrast, the controlling style refers to those teaching strategies that coerce students to think, behave, and feel in a way that is pre-determined by the teacher (Reeve et al. 2022). Originally, autonomous support was conceived as being at the opposite end of the same continuum from control; however, it has recently been shown that the dimensions are two independent variables, although correlated, and both styles can coexist in the same context (Jang et al. 2016; Reeve et al. 2022; Ryan and Deci 2017). Autonomous support not only favors positive consequences, but also cushions against negative experiences, while the controlling style facilitates maladaptive consequences and, at the same time, undermines the positive experiences (Opdenakker 2021; Reeve et al. 2022). According to the SDT (Ryan and Deci 2017; Vansteenkiste et al. 2020), the interpretation that trainee teachers have regarding the teaching styles used by their teachers will have adaptive learning consequences. On the one hand, the controlling style will reduce positive adaptive consequences (Moreno-Murcia et al. 2018), such as the SBPNs (Aelterman et al. 2019; Soenens et al. 2012), engagement (De Meyer et al. 2014) and self-confidence levels (Barlow and McCann 2019). On the other, the style supporting student autonomy will positively energize consequences such as the SBPNs (Aelterman et al. 2019), engagement (Chan et al. 2021), and self-confidence (Barlow and McCann 2019). Until recently, in order to measure the interpersonal styles of teachers in a university setting, it was necessary to use two different scales: the autonomous support scale to measure the autonomous style (Moreno-Murcia et al. 2019), and the style controller scale to measure the controlling style (Moreno-Murcia et al. 2018). In both cases, these individual scales discount some validity data, such, as convergent validity or criterion validity that are not analyzed in their validation process. We choose to select the interpersonal teaching style in higher education scale (Granero-Gallegos et al. 2021b) because both styles (i.e., autonomous support and controlling style) are measured with the same instrument that has been recently validated within the Spanish university context.

### 1.5. The Present Study

**Figure 1:**
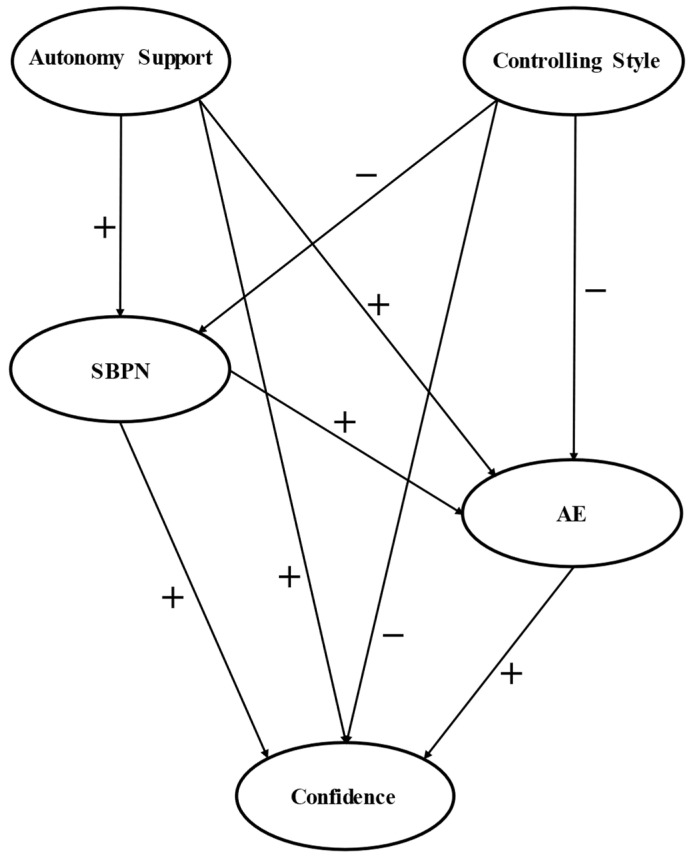
Hypothetical model with the expected relationships. Note: SBPNs = Satisfaction of basic psychological needs; AE = Academic engagement.

Given the importance of academic confidence in pre-service teachers, both during their training and later on when practicing their profession, a predictive analysis of the effects of the motivational variables and engagement on confidence is relevant and studying them should result in improvements in the initial training classes of these future teachers. In addition, to the best of our knowledge, an analysis of the mediating role of the SBPNs and academic engagement between the autonomous support and controlling teaching styles, and the confidence of pre-service teachers, has not been carried out. Therefore, the present study provides a relevant contribution to the scientific literature. Considering what the SDT postulates, and the previous studies reviewed, a hypothesized model was created (see Figure 1) to examine the above relationships. Hence, the objective of this research is to analyze the mediation of academic engagement and the SBPNs between teaching styles and confidence in teachers in initial training. The following hypotheses were established: First, autonomous support positively predicts academic confidence (H1); second, the controlling style negatively predicts academic confidence (H2); third, academic engagement and the SBPNs positively mediate the relationship between autonomous support and academic confidence (H3); fourth, academic engagement and the SPBNs negatively mediate the relationship between the controlling style and academic confidence (H4); fifth, academic engagement preceded by the SPBNs positively mediate the relationship between autonomous support and academic confidence (H5); sixth, academic engagement preceded by the SPNBs negatively mediates the relationship between the controlling style and academic confidence (H6) (Figure 1). For the study description, the strengthening the reporting of observational studies in epidemiology (STROBE) initiative (Von Elm et al. 2008) was used.

## 2. Materials and Methods

### 2.1. Design and Participants

The research design was observational, descriptive, cross-sectional, and non-randomized. Students from several Spanish universities participated. The data were collected at the end of the 2020/2021 academic year. The following inclusion criteria were established: (i) to be a student working on a master’s degree in secondary and upper secondary education teaching, vocational training and language teaching (examples of disciplines: history, mathematics, physical education, biology, economics, technology, etc.) or (ii) to be an undergraduate student in initial teacher training (i.e., early childhood education, primary education, pedagogy). The exclusion criteria established were: (i) not giving consent to the use of data in the study; or (ii) not having completed the data collection form.

### 2.2. Instruments

*Interpersonal Teaching Style in Higher Education* (EIDES). The Spanish version by Granero-Gallegos et al. (2021b) was used, comprising 11 items that measure the students’ perception of the teacher’s controlling style (6 items) (e.g., “My teacher paid less attention to students he/she disliked”) and support for teacher autonomy (5 items) (e.g., “My teacher has offered different opportunities and options during the class”). The responses were collected on a Likert scale ranging from 1 (completely disagree) to 5 (completely agree).

*Satisfaction of Basic Psychological Needs in Education* (SBPNs). The Spanish version, adapted to the academic context (León et al. 2011), of the original version by Gillet et al. (2008) was used. The instrument consists of 15 items distributed over three factors of five items each: satisfaction of autonomy (e.g., “I generally feel free to express my opinions”), competence (e.g., “I have the feeling of doing things well”), and relatedness to others (e.g., “I feel good being around the people I interact with”). The five novelty satisfaction items (e.g., “I feel I do novel things”) (González-Cutre et al. 2020) were integrated into this SBPNs scale, as suggested by the authors themselves. A Likert scale ranging from 1 (strongly disagree) to 5 (strongly agree) was used for the responses.

*Academic Engagement*. The Spanish version for students of the Utrecht Work Engagement Student Scale (UWES-SS) (Schaufeli et al. 2002) was used. The instrument consists of 17 items that make up three factors: vigor (6 items) (e.g., “In my studies I feel strong and full of energy”), dedication (5 items) (e.g., “My studies are stimulating and inspiring”), and absorption (6 items) (e.g., “I am immersed and focused on my studies”). The responses were collected on a Likert scale with a range of responses between 1 (strongly disagree) to 5 (strongly agree). Academic engagement was calculated as the average value of the scores for each of the factors that comprise it.

*Academic Confidence*. The academic confidence subscale of the Academic Self-Concept scale (ASCS) (Matovu 2014) was used, adapted to the Spanish university context (Granero-Gallegos et al. 2021a). The subscale is composed of three items (e.g., “I can follow the lectures easily”) and measures the student’s confidence in following the classes. The responses were collected on a Likert scale with a range of responses between 1 (strongly disagree) to 7 (strongly agree).

### 2.3. Procedure

The academic department heads and professors of the master’s degree in secondary education at the different universities were contacted to ask for their collaboration and informed them of the research objectives. The questionnaire was administered using an online form in which we explained the importance of the research, the anonymity of the responses, the way to fill in the scale, that participation in the study would not affect any qualification in any way, and that students could stop participating at any time. All participants consented to their inclusion in the study before participating. The study protocol was approved by the Bioethics Committee of the University of Almería (Ref:UALBIO2021/009).

### 2.4. Risk of Bias

Since the sampling was for convenience, there was no sample randomization. However, there was blinding between the participants and the researchers in charge of the data treatment and analysis. Participation was voluntary and communication with participants was conducted by email.

### 2.5. Sample Size

For the structural equation model (SEM) with five latent variables and 21 observable variables, it was calculated that a minimum of 879 subjects were needed to detect effect sizes (i.e., f2 = 0.185) with a statistical power of 0.99 and a significance level of α = 0.05. The a priori analysis of the sample size was performed with the Free Statistics Calculator v.4.0 software (Soper 2022).

### 2.6. Data Analysis

**Figure 2:**
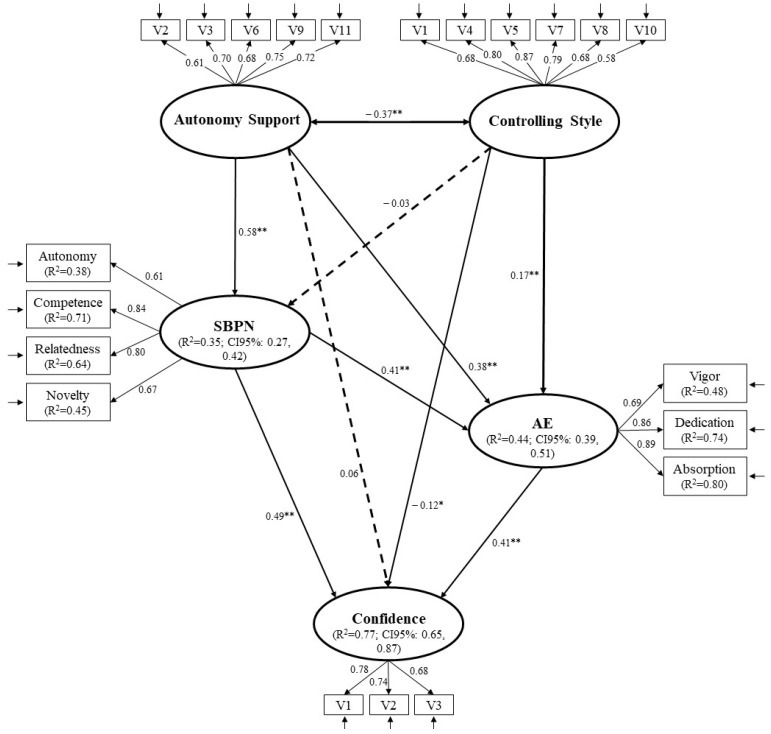
Predictive relationships between the interpersonal teaching style and academic confidence through the mediating role of the satisfaction of the basic psychological needs and academic engagement. Note: ** *p* < 0.001; * *p* < 0.01. SBPNs = Satisfaction of the basic psychological needs; AE = Academic engagement; R^2^ = Explained variance; CI = Confidence interval. The dashed lines represent non-significant relationships.

The descriptive and correlational analyses were calculated using the Statistical Package for the Social Sciences v.27 (IBM, Chicago, IL, USA) and the SEM models with AMOS v.24. The reliability of each scale was evaluated using different parameters: composite reliability (CR), ω of McDonald, and AVE (Average Variance Extracted) to measure the convergent validity. The reliability values > 0.70 and AVE values > 0.50 are considered acceptable; however, according to Hair et al. (2018), if all factorial weights are significant and greater than 0.50, then we can assume that the factors have a good convergent validity. The hypothesized predictive relationships of the teaching styles to academic confidence, with the multiple mediations of the SBPNs and academic engagement, were verified using an SEM model with latent variables. Following the proposal of Wang et al. (2017), the two-step method was used. First, the saturated model was evaluated by relating all of the constructs to each other. Second, the predictive relationships of the hypothesized model were examined. The SEM models were evaluated, taking into account the following goodness-of-fit values: the chi-square/degrees of freedom (χ^2^/gL) ratio, the CFI (comparative fit index), TLI (Tucker–Lewis Index), RMSEA (root mean square error of approximation) with its 90% confidence interval (CI), and the SRMR (standardized root mean square residual). For the χ^2^/gL ratio, values < 5.0 are considered acceptable. Tor the CFI and TLI, values > 0.90 are acceptable, and for the RMSEA and SRMR, values < 0.08 are acceptable (Hu and Bentler 1999; Marsh et al. 2004). In the SEM model, direct and indirect relationships were established between the different variables of the predictive model (see Figure 2). Given the lack of multivariate normality (Mardia’s coefficient = 75.03; *p* < 0.001), the maximum likelihood method was used with the bootstrapping procedure for 5000 re-samplings (Kline 2016). To evaluate the direct and indirect effects, the proposal of Shrout and Bolger (2002) was followed, so the indirect effects (i.e., mediated) and their 95% CI were estimated using the bootstrapping technique. The indirect effect (*p* < 0.05) was considered significant if its 95% CI did not include the zero value. Following Domínguez-Lara (2017), R^2^ was used as the effect size (ES) in order to better interpret the results.

## 3. Results

### 3.1. Participants

A total of 920 university students participated (61.8% women) from master’s programs in secondary education teaching (39.24%) and in the 3rd or 4th year of an undergraduate degree in primary education, early childhood education, and pedagogy at several Spanish universities. The age ranged from 20 to 57 years (*M* = 24.73; *SD* = 5.34). There were no missing values in the included sample data.

### 3.2. Preliminary Analysis

The descriptive statistics and correlations between the variables are presented in Table 1. The averages of the different variables are moderate and high, with the exception of the controlling style that reaches the lowest average value. Regarding the correlations, the highest, positive, and significant value is presented by the relationship of the SBPNs with academic engagement. Furthermore, the correlation of autonomous support with the SBPNs and with academic engagement is high and significant. Academic confidence presents the highest significant and positive correlation values with the SBPNs, while the relationship is negative with the controlling style. Finally, all of the variables presented an adequate reliability and AVE values. Although the AVE value for academic confidence is 0.49, it must be taken into account that, according to Hair et al. (2018), if all factorial weights are significant and greater than 0.50, then we can assume that the factors that have good convergent validity values > 0.50 are considered acceptable.

### 3.3. Main Analysis

In Step 1, the SEM model presented an acceptable goodness of fit indices: χ^2^/gL = 4.746, *p* < 0.001; CFI = 0.934; TLI = 0.919; RMSEA = 0.064 (90%CI = 0.059; 0.069), SRMR = 0.051. In Step 2, the hypothesized SEM presented a similar acceptable fit: χ^2^/gL = 4.746, *p* < 0.001; CFI = 0.934; TLI = 0.919; RMSEA = 0.064(90%CI = 0.059; 0.069), SRMR = 0.051. The relationships between the two interpersonal styles (i.e., autonomous support, controlling style) and academic confidence, as well as the relationships between the two interpersonal teaching styles and the mediators (i.e., SBPNs, academic engagement) and between the mediator and academic confidence can be seen in Figure 2 and Table 2. The explained variance was 77% for confidence, 44% for academic engagement, and 35% for the SBPNs. The SEM shows that autonomous support indirectly predicted confidence through the mediation of the SBPNs and engagement. Although both mediation relationships are weak, the mediating effect of the SBPNs between autonomous support and confidence is higher than the mediating effect of engagement. The predictive relationship between autonomous support and confidence through the SBPNs and engagement was also weak. Moreover, autonomous support does not directly predict academic confidence The controlling style positively and significantly predicts engagement and negatively predicts confidence. The SBPNs positively predicts engagement and confidence. Likewise, engagement has a positive direct effect on confidence. Engagement acted as a mediator between autonomous support and confidence and between the controlling style and confidence. Finally, Figure 2 shows the CI (95%) of R^2^ and confirms that they can be taken as measures of ES (Domínguez-Lara 2017), all of which are large (Cohen 1992).

## 4. Discussion

The objective of this study was to analyse the relationship between teacher interaction styles and academic confidence, considering the mediating role of the SBPNs and academic engagement in pre-service teachers. The main results show that autonomous support has an indirect and positive effect on confidence, highlighting the mediating role of the SBPNs above all, although the mediation of engagement is also significant. In addition, a direct and negative effect was found between the controlling style and confidence, and a positive indirect effect mediated by engagement.

In accordance with the hypothesized model, the results reveal that that autonomous support indirectly predicted academic confidence. Although both mediation relationships (i.e., SBPNs; H3) and academic engagement, preceded by the SPBNs (H5), are significant, the mediating effect of the SBPNs between autonomous support and confidence is higher than others. The findings of this study corroborate the previous research that showed the importance of establishing motivational contexts based on promoting and supporting student autonomy, as this involves an improvement in the SBPNs and leads to affective learning consequences, such as self-confidence (Reeve et al. 2018; Vansteenkiste et al. 2020). This might be because the autonomous support teaching style is a predictor that generally promotes a context supporting the satisfaction of the BPNs (*need supportive*) (Reeve et al. 2018). Thus, the BPNs play an important role in explaining the effects of educational social contexts, promoting development outcomes (Ryan and Deci 2017; Vansteenkiste et al. 2020). Specifically, the BPNs are constructs that respond to the context (Prentice et al. 2020), varying according to alterations in the context itself, while playing a mediating role between the variations in the social environment and the students’ psychosocial adjustment (Vansteenkiste et al. 2020); that is to say, the satisfaction of the BPNs also has a directional role that leads students to act on the value judgments they make about their academic abilities (Eskiler et al. 2012; Sheldon 2011; Vansteenkiste et al. 2020). Therefore, using teaching styles that promote interpersonal relationships, leads to a self-regulated approach towards satisfying the essential nutrients of the SBPNs (Aelterman et al. 2019), which according to the results of this study, increases the student’s general self-confidence (Eskiler et al. 2012) as well as their academic confidence. These aspects must be considered in the learning contexts experienced by pre-service teachers so as to improve their academic confidence.

In the present research, autonomous support does not directly predict academic confidence (H1 was rejected). These results do not coincide with other studies focused on self-confidence, such as the one by Barlow and McCann (2019); they also highlight the importance of the mediating role of cognitive elements (i.e., academic engagement) or the satisfactory motivational experiences (i.e., SBPNs) in predicting academic confidence. These results, inconsistent with what the SDT postulates, may be due to the influence that the context has on student confidence (Maclellan 2014; Vansteenkiste et al. 2020), since a context that encourages autonomous and independent learning in students, but without support guidelines, will not generate academic confidence, especially if the students have not had diverse prior experiences in this regard. Authors such as Maclellan (2014), emphasize that autonomy must be accompanied by a scaffolding of cognitive or metacognitive participation in order to develop the students’ academic confidence. However, the results of this study on pre-service teachers do highlight the importance of promoting mediation elements such as the SBPNs or engagement to generate more academic confidence.

The direct effects of the controlling style on confidence showed a negative predictive relationship (H2). According to the SDT (Ryan and Deci 2017), and the results of Barlow and McCann (2019), the use of a controlling style will move trainee teachers away from the pattern regulating their behavior, promoting negative and maladaptive consequences that will lead to a decrease in self-confidence. These results may be due to the influence that the teacher-student interaction has on student confidence (Maclellan 2014), characterized by the motivational use of rewards, the creation of organized social environments, and the intolerance towards non-teacher-led strategies (Reeve et al. 2022); that is to say, a controlling context in which trainee teachers set aside their internal motivational resources to solve their problems, and instead use a predetermined way of thinking, feeling, or behaving that has been imposed on them by the teacher, meaning they will experience a decrease in self-confidence.

The results of the present study show an indirect relationship between the controlling style and student confidence through academic engagement, but not on the SBPNs (H4 and H6 were rejected). However, the ratio is less than 0.10 (i.e., 0.07). These findings support the results of López-García et al. (under review) that show the positive relationship between the controlling style and the engagement of teachers in initial training; that is to say, using a controlling style with pre-service teachers involves the development of academic engagement and an increase in confidence. This may be due to the link between the controlling style and the use of authoritarian teaching strategies, characteristic of a more teacher-centred traditional teaching model (Soleimani 2020). In this regard, authors such as Hidalgo-Cabrillana and Lopez-Mayan (2018) demonstrate the use of conventional motivational approaches by teachers based on control and authority as a constructive and orderly way to increase prescribed behaviors (Reeve et al. 2018), thus complementing the comprehensive development of students by generating self-confidence promoted by their active engagement in the initial training process. In addition, the students are used to receiving classes that follow more traditional teaching models, so some of them need to feel that the class is organized and that their work is directed, which leads to greater academic engagement and confidence in their studies. This supports the evidence showing that both teaching styles can coexist in the same educational context (Jang et al. 2016; Reeve et al. 2022; Ryan and Deci 2017).

### 4.1. Implications for Teacher Training

The results of the present study underline the importance of the perceived motivational style adopted by pre-service teacher trainers in developing academic confidence. It is recommended that the university teaching staff themselves (i.e., trainers of future teachers) are instructed on how to apply the different interpersonal teaching styles depending on the type of students they have in their class. In this way, the teaching-learning process would be more individualized (Pascual-Arias et al. 2022). When the student profile requires a teaching profile that promotes autonomy, sessions that enhance academic engagement and feelings of competence, relatedness, autonomy, and novelty should be encouraged to improve the academic confidence of the pre-service teachers. Conversely, when the student profile requires a teaching profile in which control and authority are used, the sessions should encourage academic engagement through resilience, dedication, and concentration, and connection towards the task set, in order to increase academic confidence. To establish an autonomous support style, it is necessary to create a classroom climate based on providing learning opportunities to see and develop knowledge, which are engaging (see Appleton et al. 2008) and lead to positive psychological experiences in pre-service teachers (see Ryan and Deci 2017). As proposed by authors such as Reeve (2009), strategies should be applied that include, amongst other aspects, the use of a suitable communicative language with students (e.g., giving coherent explanations about the course objectives, contents, and learning tasks), the reasoning behind the learning tasks (e.g., explaining the underlying usefulness of the proposed activity), and establishing learning choices (e.g., giving learning options and developing knowledge around their preferences and interests). The findings of the present study suggest that these strategies by teacher trainers could promote more self-determined motivational experiences, academic engagement, and consequently, greater academic confidence during the teacher training process. However, it is also necessary to use a controlling style when the student’s profile requires it to generate confidence. Consequently, both teaching styles need to be used during the sessions.

### 4.2. Limitations and Future Prospects

Despite its contribution, there were several limitations in the present study. Firstly, the information provided by the pre-service teachers on the different variables was gathered via a questionnaire. Future lines of research should study the perception of teaching styles by addressing the responses of both the students and the teachers themselves. Secondly, the cross-sectional research design does not allow the results to be generalized. Future research should have longitudinal designs that measure the influence of motivational styles on the confidence of pre-service teachers throughout the training period in addition to using complementary observational instruments. Experimental studies can even be proposed with interventions based on different teaching styles.

## 5. Conclusions

As has been demonstrated, both interpersonal teaching styles (i.e., the controlling style and the autonomous support style) coexist in the same educational context. However, to increase academic confidence in pre-service teachers, it is necessary to generate contexts that develop the SBPNs mainly through the autonomous support style. Moreover, the study findings highlight the importance of academic engagement in pre-service teachers as a mediator between the two teaching styles and academic confidence. Accordingly, teacher trainers should incorporate these instructional styles based on autonomy and focused on increasing academic engagement to promote and maintain the quality of student learning during teacher training.

## Figures and Tables

**Table 1:** Descriptive statistics and correlations between variables.

Variable	Range	M	SD	Q1	Q2	ɷ	CR	AVE	1	2	3	4	5
Autonomous support	1–5	3.61	0.85	−0.31	−0.39	0.88	0.82	0.52	-	−0.29 **	0.50 **	0.52 **	0.37 **
Controlling style	1–5	2.28	0.96	0.52	−0.41	0.93	0.88	0.55		-	−0.16 **	−0.06	−0.25 **
SBPNs	1–5	3.72	0.67	0.09	−0.39	0.82	0.79	0.51			-	0.60 **	0.46 **
Academic Engagement	1–5	3.45	0.82	−0.28	−0.09	0.86	0.77	0.52				-	0.38 **
Academic Confidence	1–7	5.63	10.06	−0.64	−0.16	0.75	0.72	0.49					-

Note. ** The correlation is significant at the 0.01 level; * The correlation is significant at the 0.05 level; M = Mean; SD = Standard Deviation; SBPNs = Satisfaction of Basic Psychological Needs; Q1 = Skewness; Q2 = Kurtosis; ɷ = Omega of McDonald; CR = Composite Reliability; AVE = Average Variance Extracted.

**Table 2:** Estimation of significant standardized parameters and statistics of the mediation model.

Independent Variable	Dependent Variable	Mediator	β	SE	95% CI	
					Inf	Sup
Direct effects						
Autonomous support	SBPNs		0.58 **	0.05	0.49	0.64
Autonomous support	Academic engagement		0.38 **	0.06	0.29	0.46
Controlling style	Academic engagement		0.17 **	0.05	0.09	0.23
Controlling style	Academic confidence		−0.12 *	0.06	−0.24	−0.06
SBPNs	Academic engagement		0.41 **	0.08	0.32	0.49
SBPNs	Academic confidence		0.49 **	0.10	0.37	0.61
Academic engagement	Academic confidence		0.41 **	0.06	0.31	0.47
Indirect effects						
Autonomous support	Academic engagement	SBPNs	0.24 **	0.03	0.17	0.29
Autonomous support	Academic confidence	SBPNs	0.28 **	0.04	0.19	0.34
Autonomous support	Academic confidence	Academic engagement	0.16 **	0.04	0.05	0.27
Controlling style	Academic confidence	Academic engagement	0.07	0.02	−0.06	0.14

Note. β = Estimation of standardized parameters; SE = standard error; 95% CI = 95% confidence interval; Inf = Lower limit of 95% CI; Sup = Upper limit of 95% CI; ** *p* < 0.01; * *p* < 0.05.

## Data Availability

The data presented in this study are available upon request from the corresponding author. The data are not publicly available due to privacy.

## References

[B1-jintelligence-10-00071] Adeshola Brahim, Agoyi Mary (2022). Examining factors influencing e-learning engagement among university students during COVID-19 pandemic: A mediating role of “learning persistence”. Interactive Learning Environments.

[B2-jintelligence-10-00071] Aelterman Nathalie, Vansteenkiste Maarten, Haerens Leen, Soenens Bart, Fontaine Johnny R.J., Reeve Johnmarshall (2019). Toward an integrative and fine-grained insight in motivating and demotivating teaching styles: The merits of a circumplex approach. Journal of Educational Psychology.

[B3-jintelligence-10-00071] Akbari Omidullah, Sahibzada Javed (2020). Students’ Self-Confidence and Its Impacts on Their Learning Process. American International Journal of Social Science Research.

[B4-jintelligence-10-00071] Amerstorfer Carmen M., Freiin von Münster-Kistner Clara (2021). Student Perceptions of Academic Engagement and Student-Teacher Relationships in Problem-Based Learning. Frontiers in Psychology.

[B5-jintelligence-10-00071] Appleton James. J., Christenson Sandra L., Furlong Michael J. (2008). Student engagement with school: Critical conceptual and methodological issues of the construct. Psychology in the Schools.

[B6-jintelligence-10-00071] Bakadorova Olga, Lazarides Rebecca, Raufelder Diana (2020). Effects of social and individual school self-concepts on school engagement during adolescence. European Journal of Psychology of Education.

[B7-jintelligence-10-00071] Barlow Alexis, McCann Margaret (2019). Academic self-confidence: Students progressing from further to higher education. EDULEARN19 Proceedings.

[B8-jintelligence-10-00071] Burgueño Rafael, González-Cutre David, Sicilia Álvaro, Alcaraz-Ibáñez Manuel, Medina-Casaubón Jesús (2021). Is the instructional style of teacher educators related to the teaching intention of pre-service teachers? A Self-Determination Theory perspective-based analysis. Educational Review.

[B9-jintelligence-10-00071] Buzzai Caterina, Sorrenti Luana, Costa Sebastiano, Toffle Mary Ellen, Filippello Pina (2021). The relationship between school-basic psychological need satisfaction and frustration, academic engagement and academic achievement. School Psychology International.

[B10-jintelligence-10-00071] Chan Sokhom, Maneewan Sorakrich, Koul Ravinder (2021). Teacher educators’ teaching styles: Relation with learning motivation and academic engagement in pre-service teachers. Teaching in Higher Education.

[B11-jintelligence-10-00071] Chang Jen-Chia-Tu-Tai Wu, Ye Jhen-Ni (2022). A Study of Graduate Students’ Achievement Motivation, Active Learning, and Active Confidence Based on Relevant Research. Frontiers in Psychology.

[B12-jintelligence-10-00071] Cohen Jacob (1992). A power primer. Psychological Bulletin.

[B13-jintelligence-10-00071] De Meyer Jotie, Tallir Isabel B., Soenens Bart, Vansteenkiste Maarten, Aelterman Nathalie, Berghe Lynn Van den, Speleers Lise, Haerens Leen (2014). Does observed controlling teaching behavior relate to students’ motivation in physical education?. Journal of Educational Psychology.

[B14-jintelligence-10-00071] Demanet Jannick, Van Houtte Mieke, Demanet Jannick, Van Houtte Mieke (2019). School Effects on Deviance: An International Perspective. Resisting Education: A Cross-National Study on Systems and School Effects.

[B15-jintelligence-10-00071] DeVito Maria (2016). Factors Influencing Student Engagement. Unpublished Certificate of Advanced Study Thesis.

[B16-jintelligence-10-00071] Domínguez-Lara Sergio A. (2017). Magnitud del efecto en análisis de regresión. Interacciones: Revista de Avances en Psicología.

[B17-jintelligence-10-00071] Engels Nadine, Aelterman Antonia, Van Petegem Karen, Schepens Annemie (2004). Factors which influence the well-being of pupils in Flemish secondary schools. Educational Studies.

[B18-jintelligence-10-00071] Eskiler Ersin, Sari Ihsan, Soyer Fikret, Talaghir Laurentiu (2012). The Effect of basic psychological needs on university students’ self-confidence. Annals of “Dunarea de Jos” University of Galati. Fascicle XV, Physical Education and Sport Management 1.

[B19-jintelligence-10-00071] Gao Qiyang, Bao Chenye, Du Haiping, Yan Ruixiang (2021). The mediating role of basic psychological needs satisfaction in the relationship between teacher-student relationships and academic engagement in China. Asia Pacific Journal of Education.

[B20-jintelligence-10-00071] Gilbert William, Guay Frédéric, Morin Alexandre J. S. (2022). Can teachers’ need-supportive practices moderate the big-fish-little-pond effect? A quasi-experimental study with elementary school children. Contemporary Educational Psychology.

[B21-jintelligence-10-00071] Gillet Nicolas, Rosnet Elisabeth, Vallerand Robert J. (2008). Développement d’une échelle de satisfaction des besoins fondamentaux en contexte sportif. Canadian Journal of Behavioural Science.

[B22-jintelligence-10-00071] González-Cutre David, Romero-Elías María, Jiménez-Loaisa Alejandro, Beltrán-Carrillo Vicente J., Hagger Martin S. (2020). Testing the need for novelty as a candidate need in basic psychological needs theory. Motivation and Emotion.

[B23-jintelligence-10-00071] Granero-Gallegos Antonio, Baena-Extremera Antonio, Escaravajal Juan Carlos, Baños Raúl (2021a). Validation of the Academic Self-Concept Scale in the Spanish university context. Education Sciences.

[B24-jintelligence-10-00071] Granero-Gallegos Antonio, Hortigüela-Alcalá David, Hernando-Garijo Alejandra, Carrasco-Poyatos María (2021b). Estilo docente y competencia en educación superior: Mediación del clima motivacional. Educación XX1.

[B25-jintelligence-10-00071] Hair Joseph F., Black William C., Babin Barry J., Anderson Rolph E. (2018). Multivariate Data Analysis.

[B26-jintelligence-10-00071] Hidalgo-Cabrillana Ana, Lopez-Mayan Cristina (2018). Teaching styles and achievement: Student and teacher perspectives. Economics of Education Review.

[B27-jintelligence-10-00071] Hu Li Tze, Bentler Peter M. (1999). Cutoff criteria for fit indexes in covariance structure analysis: Conventional criteria versus new alternatives. Structural Equation Modeling.

[B28-jintelligence-10-00071] Ireson Judith, Hallam Susan (2009). Academic self-concepts in adolescence: Relations with achievement and ability grouping in schools. Learning and Instruction.

[B29-jintelligence-10-00071] Jang Hyungshim, Kim Eun Joo, Reeve Johnmarshall (2016). Why students become more engaged or more disengaged during the semester: A self-determination theory dual-process model. Learning and Instruction.

[B30-jintelligence-10-00071] Kahu Ella R. (2013). Framing student engagement in higher education. Studies in Higher Education.

[B31-jintelligence-10-00071] Kline Rex B. (2016). Principles and Practice of Structural Equation Modeling.

[B32-jintelligence-10-00071] León Jaime, Domínguez Evelia, Núñez José L., Pérez Araceli, Martín-Albo José (2011). Traducción y validación de la versión española de la Échelle de Satisfacción des Besoins Psychologiques en el contexto educativo. Anales de Psicologia.

[B33-jintelligence-10-00071] Li Jing, Zhang Na, Yao Meilin, Xing Huilin, Liu Hongrui (2021). Academic Social Comparison and Depression in Chinese Adolescents: The Mediating Role of Basic Psychological Needs Satisfaction. School Mental Health.

[B34-jintelligence-10-00071] Lu Min, Walsh Kerryann, White Sonia, Shield Paul (2017). The Associations between Perceived Maternal Psychological Control and Academic Performance and Academic Self-Concept in Chinese Adolescents: The Mediating Role of Basic Psychological Needs. Journal of Child and Family Studies.

[B35-jintelligence-10-00071] Maclellan Effie (2014). How might teachers enable learner self-confidence? A review study. Educational Review.

[B36-jintelligence-10-00071] Malandrakis George (2018). Influencing Greek pre-service teachers’ efficacy beliefs and self-confidence to implement the new ‘Studies for the Environment’ curricula. Environmental Education Research.

[B37-jintelligence-10-00071] Manzano-Sánchez David, Valero-Valenzuela Alfonso, Hortigüela-Alcalá David (2021). Sistema Educativo y actuación ante la pandemia de la COVID-19: Opinión y perspectivas de mejora según los docentes. Revista Española de Educación Comparada.

[B38-jintelligence-10-00071] Marsh Herbert W., Hau Kit Tai, Wen Zhonglin (2004). In search of golden rules: Comment on hypothesis-testing approaches to setting cutoff values for fit indexes and dangers in overgeneralizing Hu and Bentler’s (1999) findings. Structural Equation Modeling.

[B39-jintelligence-10-00071] Martins Izaaias, Monsalve Juan Pablo Pérez, Martinez Andrés Velasquez (2018). Self-confidence and fear of failure among university students and their relationship with entrepreneurial orientation: Evidence from Colombia. Academia Revista Latinoamericana de Administracion.

[B40-jintelligence-10-00071] Matovu Musa (2014). A Structural Equation Modelling of the Academic Self-Concept Scale. International Electronic Journal of Elementary Education.

[B41-jintelligence-10-00071] Meeus Wil, Cools Wouter, Placklé Inge (2018). Teacher educators developing professional roles: Frictions between current and optimal practices. European Journal of Teacher Education.

[B42-jintelligence-10-00071] Moreno-Murcia Juan Antonio, Huéscar Elisa, Pintado Román, Marzo Juan Carlos (2019). Diseño y validación de la escala de apoyo a la autonomía en educación superior: Relación con la competencia laboral del discente. REOP- Revista Española De Orientación y Psicopedagogía.

[B43-jintelligence-10-00071] Moreno-Murcia Juan Antonio, Pintado Román, Huéscar Elisa, Marzo Juan Carlos (2018). Estilo interpersonal controlador y percepción de competencia en educación superior. European Journal of Education and Psychology.

[B44-jintelligence-10-00071] Muñiz-Rodríguez Laura, Velázquez Pedro Alonso, Muñiz Luis J. Rodríguez, Valcke Martín (2016). ¿Hay un vacío en la formación inicial del profesorado de matemáticas de Secundaria en España respecto a otros países?. Revista de Educacion.

[B45-jintelligence-10-00071] Muñiz-Rodríguez Laura, Velázquez Pedro Alonso, Rodríguez-Muñiz Luis J., Valcke Martín (2021). Are secondary mathematics student teachers ready for the profession? A multi-actor perspective on mathematics student teachers’ mastery of related competences. Advances in Intelligent Systems and Computing, paper presented at ICEUTE 2020: The 11th International Conference on EUropean Transnational Educational (ICEUTE 2020), Burgos, Spain, September 16–18.

[B46-jintelligence-10-00071] OECD (2014). New Insights from TALIS 2013. Talis.

[B47-jintelligence-10-00071] Opdenakker Marie-Chirstine (2021). Need-Supportive and Need-Thwarting Teacher Behavior: Their Importance to Boys’ and Girls’ Academic Engagement and Procrastination Behavior. Frontiers in Psychology.

[B48-jintelligence-10-00071] Parker Wendy, Donato Kirsten, Cardone Katie, Cerulli Jennifer (2017). Experiential Education Builds Student Self-Confidence in Delivering Medication Therapy Management. Pharmacy.

[B49-jintelligence-10-00071] Pascual-Arias Cristina P., López-Pastor Víctor M., Alcalá David Hortigüela (2022). Student participation in the assessment and in-service teacher education as a tool for transparency and improvement of education quality. Espiral. Cuadernos del profesorado.

[B50-jintelligence-10-00071] Perry Patricia (2011). Concept analysis: Confidence/self-confidence. Nursing Fórum.

[B51-jintelligence-10-00071] Ping Cui, Schellings Gonny, Beijaard Dowe (2018). Teacher educators’ professional learning: A literature review. Teaching and Teacher Education.

[B52-jintelligence-10-00071] Prentice Mike, Jayawickreme Eranda, Fleeson William (2020). An experience sampling study of the momentary dynamics of moral, autonomous, competent, and related need satisfactions, moral enactments, and psychological thriving. Motivation and Emotion.

[B53-jintelligence-10-00071] Pulford Briony D., Woodward Bethan, Taylor Eve (2018). Do social comparisons in academic settings relate to gender and academic self-confidence?. Social Psychology of Education.

[B54-jintelligence-10-00071] Reeve Johnmarshall (2009). Why teachers adopt a controlling motivating style toward students and how they can become more autonomous supportive. Educational Psychologist.

[B55-jintelligence-10-00071] Reeve Johnmarshall, Jang Hye-Ryen, Jang Hyungshim (2018). Personality-based antecedents of teachers’ autonomy-supportive and controlling motivating styles. Learning and Individual Differences.

[B56-jintelligence-10-00071] Reeve Johnmarshall, Ryan Richard M., Cheon Sung Hyeon, Matos Lennia, Kaplan Haya (2022). Supporting Students’ Motivation.

[B57-jintelligence-10-00071] Reeve Johnmarshall, Cheon Sung Hyeon, Jang Hye-Ryen (2019). A teacher-focused intervention to enhance students’ classroom engagement. Handbook of Student Engagement Interventions: Working with Disengaged Students.

[B58-jintelligence-10-00071] Ryan R. M., Deci Edward L., Vansteenkiste Maarten, Soenens Bart (2021). Building a science of motivated persons: Self-determination theory’s empirical approach to human experience and the regulation of behavior. Motivation Science.

[B59-jintelligence-10-00071] Ryan Richard M., Deci Edward L. (2017). Self-Determination Theory: Basic Psychological Needs in Motivation, Development, and Wellness.

[B60-jintelligence-10-00071] Sadler Ian (2013). The role of self-confidence in learning to teach in higher education. Innovations in Education and Teaching International.

[B61-jintelligence-10-00071] Sánchez-Cabrero Roberto, Pericacho-Gómez Francisco Javier (2022). Profile and perceptions of the students of the Master in secondary education teacher training in Spain. Espiral. Cuadernos del Profesorado.

[B62-jintelligence-10-00071] Sander Paul, Sanders Lalage (2005). Giving Presentations: The impact on students’ perceptions. Psychology Teaching Review.

[B63-jintelligence-10-00071] Schaufeli Wilmar B., Bakker Arnold B., Salanova Marisa (2006). The measurement of work engagement with a short questionnaire: A cross-national study. Educational and Psychological Measurement.

[B64-jintelligence-10-00071] Schaufeli Wilmar B., Martínez Isabe. M., Pinto Alexandra Marquez, Salanova Marisa, Barker Arnold B. (2002). Burnout and engagement in university students a cross-national study. Journal of Cross-Cultural Psychology.

[B65-jintelligence-10-00071] Sharma Divya (2016). How Confident are our Pre Service Teachers of Primary Training Colleges—A Peep into the Effect of Academic Performance on the Confidence Levels. Asian Journal of Research in Social Sciences and Humanities.

[B66-jintelligence-10-00071] Sheldon Kennon M. (2011). Integrating Behavioral-Motive and Experiential-Requirement Perspectives on Psychological Needs: A Two Process Model. Psychological Review.

[B67-jintelligence-10-00071] Shrout Patrick E., Bolger Nial (2002). Mediation in experimental and nonexperimental studies: New procedures and recommendations. Psychological Methods.

[B68-jintelligence-10-00071] Soenens Bart, Sierens Eline, Vansteenkiste Maarten, Dochy Filip, Goossens Luc (2012). Psychologically controlling teaching: Examining outcomes, antecedents, and mediators. Journal of Educational Psychology.

[B69-jintelligence-10-00071] Soleimani Neda (2020). ELT teachers’ epistemological beliefs and dominant teaching style: A mixed method research. Asian-Pacific Journal of Second and Foreign Language Education.

[B70-jintelligence-10-00071] Soper Daniel S. (2022). A-priori Sample Size Calculator for Student *t*-Tests—Free Statistics Calculators. https://bit.ly/3oxyIQS.

[B71-jintelligence-10-00071] Sumantri Mohamad Syarif, Prayuningtyas Angger W., Rachmadtullah Reza, Magdalena Ina (2018). The Roles of Teacher-Training Programs and Student Teachers’ Self-Regulation in Developing Competence in Teaching Science. Advanced Science Letters.

[B72-jintelligence-10-00071] Vansteenkiste Maarten, Ryan Richard M., Soenens Bart (2020). Basic psychological need theory: Advancements, critical themes, and future directions. Motivation and Emotion.

[B73-jintelligence-10-00071] Von Elm Erik, Altman Douglas G., Egger Mattias, Pocock Stuart J., Gøtzsche Peter C., Vandenbrouckef Jan P. (2008). The Strengthening the Reporting of Observational Studies in Epidemiology (STROBE) Statement: Guidelines for reporting observational studies. Bulletin of the World Health Organization.

[B74-jintelligence-10-00071] Wang Jichuan, Hefetz Amir, Liberman Gabriel (2017). Applying structural equation modelling in educational research. Culture and Education.

